# Natural History of Treated Subarachnoid Neurocysticercosis

**DOI:** 10.4269/ajtmh.19-0436

**Published:** 2019-10-21

**Authors:** Theodore E. Nash, Elise M. O’Connell, Dima A. Hammoud, Lauren Wetzler, JeanAnne M. Ware, Siddhartha Mahanty

**Affiliations:** 1 Laboratory of Parasitic Diseases, National Institute of Allergy and Infectious Diseases, National Institutes of Health, Bethesda, Maryland;; 2 Center for Infectious Disease Imaging, Clinical Center, National Institutes of Health, Bethesda, Maryland

## Abstract

Subarachnoid neurocysticercosis (SUBNCC) is usually caused by an aberrant proliferative form of *Taenia solium* causing mass effect and arachnoiditis. Thirty of 34 SUBNCC patients were treated with extended cysticidal and anti-inflammatory regimens and followed up a median of 4.2 years posttreatment (range: 15 for ≥ 4 years, 20 ≥ 2 years, 26 > 1 year, and 3 < 1 year). The median ages at the time of first symptom, diagnosis, and enrollment were 29.7, 35.6, and 37.9 years, respectively; 58.8% were male and 82.4% were Hispanic. The median time from immigration to symptoms (minimum incubation) was 10 years and the estimated true incubation period considerably greater. Fifty percent also had other forms of NCC. Common complications were hydrocephalus (56%), shunt placement (41%), infarcts (18%), and symptomatic spinal disease (15%). Thirty patients (88.2%) required prolonged treatment with albendazole (88.2%, median 0.55 year) and/or praziquantel (61.8%; median 0.96 year), corticosteroids (88.2%, median 1.09 years), methotrexate (50%, median 1.37 years), and etanercept (34.2%, median 0.81 year), which led to sustained inactive disease in 29/30 (96.7%) patients. Three were treated successfully for recurrences and one has continuing infection. Normalization of cerebral spinal fluid parameters and cestode antigen levels guided treatment decisions. All 15 patients with undetectable cestode antigen values have sustained inactive disease. There were no deaths and moderate morbidity posttreatment. Corticosteroid-related side effects were common, avascular necrosis of joints being the most serious (8/33, 24.2%). Prolonged cysticidal treatment and effective control of inflammation led to good clinical outcomes and sustained inactive disease which is likely curative.

## INTRODUCTION

Neurocysticercosis (NCC) is caused by infection of the brain with metacestodes or cysts of the pork tapeworm, *Taenia solium*. Signs and symptoms are variable depending on cyst location and number, degree of inflammation, and parasite form. Infection of the brain parenchyma, ventricles, and subarachnoid space typically causes distinctive clinical manifestations. Cysts lodged within the brain parenchyma, or within the sulci of the subarachnoid space partially abutting the brain parenchyma, mostly cause seizures. By contrast, cysts lodged within the ventricular system obstruct cerebral spinal fluid (CSF) flow with or without associated ventriculitis, resulting in hydrocephalus. Multiple compartments of the brain are frequently involved simultaneously.^1,2^

Subarachnoid NCC (SUBNCC) is the most serious form of NCC and differs from other types of involvement because it is an aberrant proliferating parasite derived from *T. solium* cysts.^3–5^ Although mostly located in the subarachnoid spaces, it rarely involves the ventricles. Compared with normal structured cysts*,* which have the anatomic structure of a cysticercus type of larva with the scolex fully invaginated into its fluid-containing bladder, SUBNCC consists of unstructured growth of disorganized cyst elements (usually lacking a scolex), which may or may not demonstrate multiple fluid-filled vesicles resembling a “bunch of grapes” or racemose cysts.^3,4,6–8^ Because cystic vesicular structures degenerate to arachnoiditis (enhancement by MRI examination) directed to cyst walls with or without prior anthelminthic treatment or may not have been able to fully form, the presence of vesicular cystic structures are not required to define SUBNCC. Depending on the series and case definition, which many times include ventricular involvement, the prevalence of subarachnoid disease varies,^9–11^ but in a large and well-defined series in the United States made up about 20% of unselected cases.^12^

In its most advanced form, multiple brain cisterns are enlarged causing varying degrees of mass effects. When cystic enlargements predominate, inflammation (manifested as enhancement on MRI) is usually limited^6,13^ but with progression or cysticidal treatment, acute and chronic arachnoiditis ensues directed toward degenerating cysts and residual antigen.^6^ Long-standing Inflammation is responsible for many of the severe and often fatal complications of SUBNCC, including hydrocephalus, focal brain damage, nerve entrapments, and infarcts.^2,7^ There are no randomized treatment trials and no studies to determine effectiveness and best use of cysticidal drugs, corticosteroids, duration of treatment, or the utility of other immunosuppressive medications. Moreover, there are no accepted end points that predict treatment efficacy and probability of recurrence. Investigators base their treatment decisions on their individual preference and experience as well as pragmatic considerations such as cost and availability of medications, or avoidance of the side effects of corticosteroids.^14^

Here, we report the clinical course and outcome of 34 patients diagnosed with SUBNCC enrolled at the NIH in an observational study. Treatment consisted of long-term cysticidal drugs to kill cysts accompanied by suppression of the host inflammatory response. Intensity and duration of treatments, guided by improvement in CSF parameters and cestode antigen levels, resulted in no mortality, good clinical outcome, and sustained inactive disease in all but one patient.

## METHODS

### Patients included and treatment approach.

This series is a retrospective review of all patients enrolled with SUBNCC in the Laboratory of Parasitic Diseases protocol, NIH (85-I-0127), between March 1985 and January 2019. Although treatments were not mandated in the protocol, some procedures and testing were performed on every patient. All but one was enrolled after 2000 and each fulfilled the criteria^15^ for the diagnosis of NCC, including consistent or diagnostic imaging, positive Western blot for *T. solium* antibodies, expected clinical course, and response to treatment, when given. Patients were consented and enrolled into protocol Natioinal Institutes of Health (NIH) protocol 85-I-0127 (a natural history protocol allowing evaluation, treatment, and follow-up of patients with NCC), approved by the National Institutes of Allergy and Infectious Diseases Institutional Review Board. All patients were referred, usually with a proven or likely diagnosis of NCC and a majority had treatment initiated before enrollment.

SUBNCC includes any of the following imaging patterns: involvement of single or multiple subarachnoid spaces or cisterns with unilocular or multivesicular cystic vesicular structures commonly causing localized enlargement and displacement of adjacent structures, substantial subarachnoid-located enhancement, diffuse enhancement of the meninges, large amorphous calcification(s), or uncommonly diagnostic histopathology. Any of the above associated with normal structured cysts, usually with a scolex located within the subarachnoid spaces is not considered SUBNCC. Treatment decisions were based on the medical needs and clinical state of each patient and preferentially consisted of albendazole or if required, praziquantel along with initial high-dose corticosteroids (10–16 mg dexamethasone/day). Cimetidine was sometimes used to boost serum praziquantel levels. Combination treatment evolved into the preferred treatment and usually consisted of albendazole (15 mg/kg day) and praziquantel (50–60 mg/kg/day) in divided doses with food. In some treatment-refractory cases, we used maximum doses of albendazole at 30 mg/kg/day and/or praziquantel at 100 mg/kg/day. The corticosteroid-sparing/replacement anti-inflammatory drugs methotrexate^15,16^ and/or etanercept^17^ (an α-tumor necrosis factor inhibitor) were used as previously described.^17^ MR imaging was obtained shortly after enrollment and otherwise to evaluate clinical changes or treatment effects. Imaging was obtained minimally every 3 months while on therapy to assess treatment-related changes, including cyst degeneration, the presence and extent of inflammation (degree and pattern of enhancement), and other indicative changes. Patients were closely followed up, usually weekly for the first 1–2 months. Lumbar punctures (LPs) for evaluation of CSF parameters were initially performed as clinically indicated, but as the usefulness of CSF changes became apparent, they were routinely requested approximately every 3 months. Undetectable or near-undetectable levels of CSF cestode antigen were considered an optimal response and a reason to stop treatment. A dexamethasone taper was initiated when the patient was clinically stable (usually after 7–10 days) at 1 mg/week dose reduction until about 3 mg/day was reached, at which time prednisone was substituted at the equivalent dose (20 mg/day) and then decreased by 1 mg/week. Initially, both methotrexate and etanercept were administered at lower doses of less than 20 mg/week orally and 25 mg/week subcutaneously, respectively, but increased to 20 mg/week methotrexate with folic acid on other weekdays and 50 mg etanercept/week after they were found to be safe in this population. Usual prophylactic medications and precautions for infections in immunocompromised hosts were used routinely as medically indicated. In patients with substantial and continued central nervous system (CNS) inflammation, methotrexate and/or etanercept were continued in the absence of cysticidal medication. A few patients with recurrences were initially retreated with low-dose corticosteroids or other immunosuppressive medications that were rapidly tapered off while maintained on high-dose cysticidal medication(s). After all treatments were stopped, the patients were followed up as clinically indicated, minimally semi-annually and then annually indefinitely. We performed serial LPs for CSF analyses, whenever possible, following cessation of treatment to monitor for relapse in patients with complicated hard-to-treat disease, patients in whom CSF parameters did not totally normalize, and patients whose MRIs continued to show cystic enlargement but with improved CSF parameters.

### Cestode antigen assays.

Most cestode antigen analyses were performed using a Luminex bead–based assay (T. Nutman, unpublished data) at the same time and using a single batch of reagents supplied by Dr. P. Dorny (Department of Biomedical Sciences, Prince Leopold Institute of Tropical Medicine, Antwerp, Belgium).^18^ More recently, a cysticercosis antigen ELISA commercial kit (ApDia, Advanced Practical Diagnostics bvda, Turnhout, Belgium) that used the same monoclonal antibodies was used. Standard curves using *T. solium* antigen allowed conversion of optical density to antigen concentration of ng/mL and comparison between assays run on different days. The limit of detection of both assays was < 3 ng/mL. Serial cestode antigen values were determined in most patients using the Luminex assay using the same batch of reagents.

### Magnetic resonance imaging changes.

Patients undergoing imaging underwent a non-enhanced computer tomographic (CT) imaging on enrollment if not recently performed and available for review. MR scans were either performed at the NIH using a 1.5T or 3T Philips (Koninklijke Philips N.V) or Siemens MR (Siemans AG) scanner or at other facilities using a variety of different scanners. Gadolinium-based contrast material was used to assess enhancement in most studies. All the scans included at least T1, T2, fluid-attenuated inversion recovery (FLAIR), diffusion-weighted, and enhanced T1-weighted sequences. More recent scans at the NIH also included postcontrast axial 2D FLAIR images, postcontrast sagittal 3D T1 fast field echo (FFE) scans, susceptibility-weighted imaging to detect calcifications, and fast imaging employing steady-state acquisition or balanced FFE, which provide high signal-to-noise ratio, strong signal from fluid, and low signal from the parenchyma resulting in good contrast between the CSF and brain. Earlier imaging was less inclusive and expanded as the technology improved.

## RESULTS

### Illustrative cases.

#### Illustrative case A.

**Figure 1.**
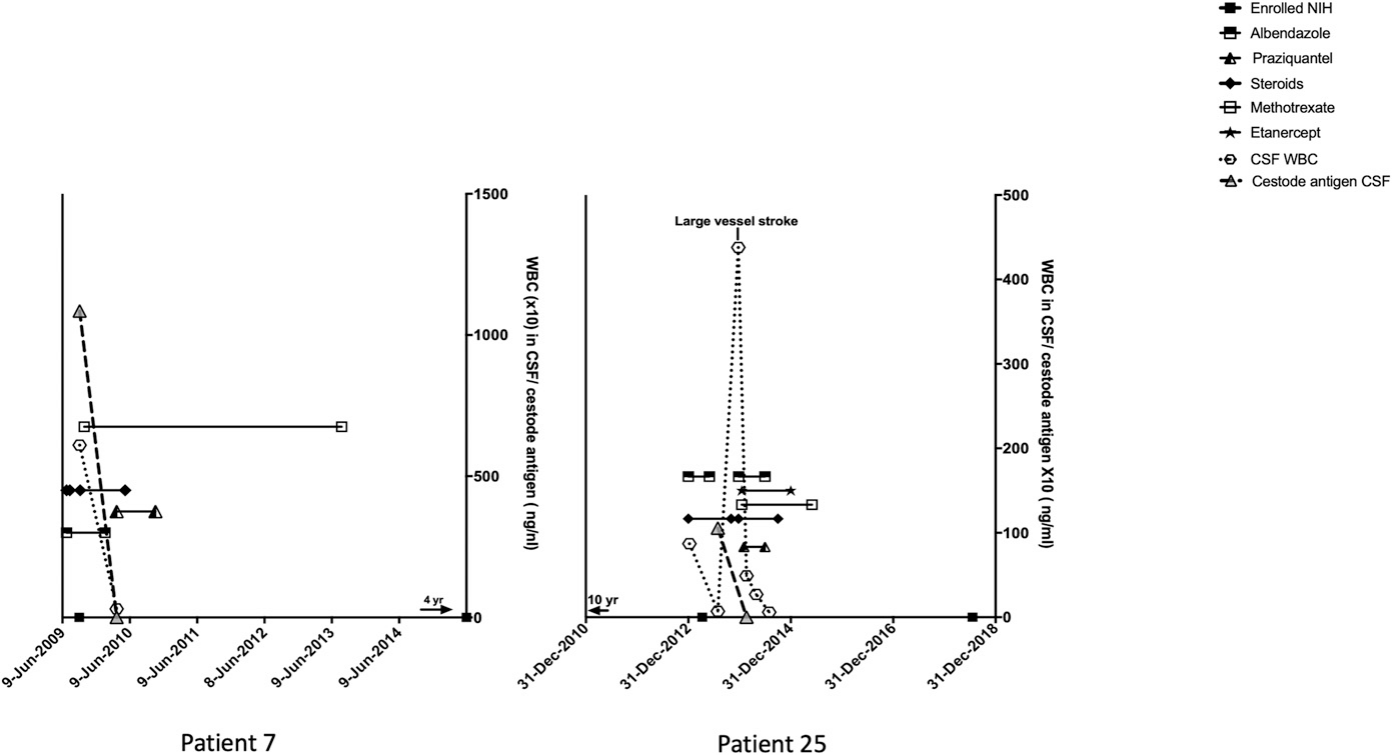
Course of disease, NIH enrollment, drug administration and cerebral spinal fluid (CSF) white cell blood count (WBC) counts, and cestode antigen levels over time. Small arrows above the *x* axis indicate the extent of time before or after labeled dates.

**Figure 2.**
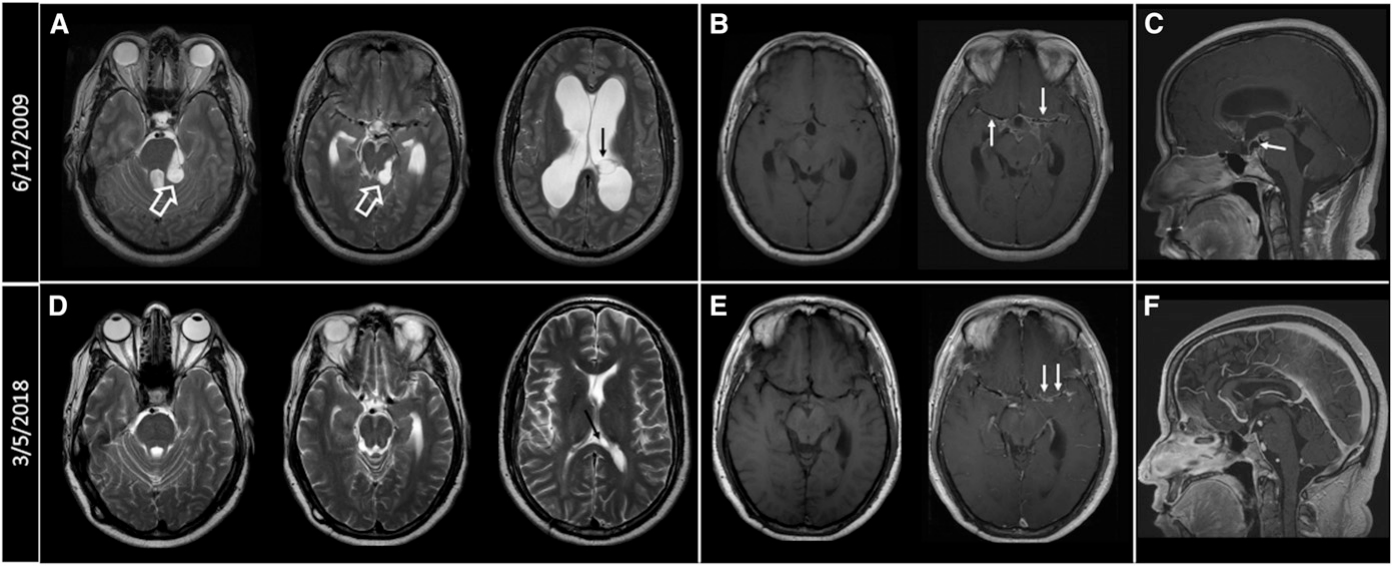
(**A**) Axial T2-weighted images at the levels of the pons, midbrain, and lateral ventricles. Cystic masses are seen in the perimesencephalic cistern (white hollow arrows) with secondary distortion of adjacent brain stem structures. Hydrocephalus is noted with transependymal cerebrospinal fluid seepage. A left lateral ventricular cyst can also be seen (black arrow). (**B**) Pre- and postcontrast T1-weighted images showing abnormal enhancement along the courses of the middle cerebral arteries (MCAs) and anterior cerebral arteries (ACAs) and extending into the interpeduncular cistern (white arrows). (**C**) Sagittal postcontrast T1-weighted images showing suprasellar rim enhancing cyst anteriorly displacing the pituitary stalk (white arrow). (**D**) Axial T2-weighted images obtained on March 5, 2018, showing resolution of cystic masses and mass effect on the brain stem structures. The ventricular cyst has markedly decreased in size (black arrow). (**E**) Pre- and postcontrast T1-weighted images show significant improvement of the perivascular and meningeal enhancing lesions (white arrows). (**F**) Sagittal postcontrast T1-weighted images show resolution of the suprasellar cyst.

Patient 7 (Figures 1 and 2) is a Hispanic male who immigrated from Honduras in 1983. He was well until 2003 when he experienced headaches, blurry vision, and then unconsciousness with incontinence. He recovered spontaneously without treatment; no cause was determined after a cursory evaluation. In 2005, he developed increasingly severe headaches that culminated when he was found unconscious on June 11, 2009. MRI showed hydrocephalus, extensive SUBNCC, including cystic involvement of the basilar cisterns and a left lateral ventricular cyst (Figure 2A), irregular calcifications and vessel wall irregularities of both middle cerebral arteries within the Sylvian fissures (Figure 2B), and a suprasellar cyst anteriorly displacing the pituitary stalk (Figure 2C). He had an initial complicated course after he was started on albendazole with corticosteroids, including placement of a ventricular peritoneal shunt. On evaluation at the NIH on September 8, 2009, he complained of episodes of double vision and generalized weakness, and neurological examination revealed a positive Romberg test. Lumbar puncture on September 10, 2009, showed 109 red blood cell counts (RBCs)/mm^3^, 61 white blood cell counts (WBCs)/mm^3^ with 70% lymphocytes, 444 mg/dL protein, 35 mg/dL glucose, and the presence of cestode antigen. He was started on dexamethasone, methotrexate for corticosteroid sparing on October 5, 2009, and albendazole. His course was complicated by numerous medical problems, including recurrent pulmonary emboli, diagnosed on admission, severe corticosteroid-induced side effects, including weight gain, massive lower extremity edema, diabetes, and bilateral avascular necrosis of the hips, not requiring surgical intervention. Albendazole was stopped on January 15, 2010, and eventually praziquantel substituted on March 30, 2010. Lumbar puncture on that date showed 3 WBCs/mm, 3 248 mg/dL protein, 43 mg/dL glucose, and an undetectable cestode antigen. His clinical condition and MR imaging continued to improve on therapy, and treatments with corticosteroid and praziquantel were stopped in March and July of 2010, respectively, and methotrexate in August of 2013. Subsequently, he developed asymptomatic enlargement of the left lateral ventricle caused by an entrapped degenerated cyst that could not be removed earlier. Serial MRIs documented stable resolved changes; he has been asymptomatic for 9 years.

#### Illustrative case B.

**Figure 3.**
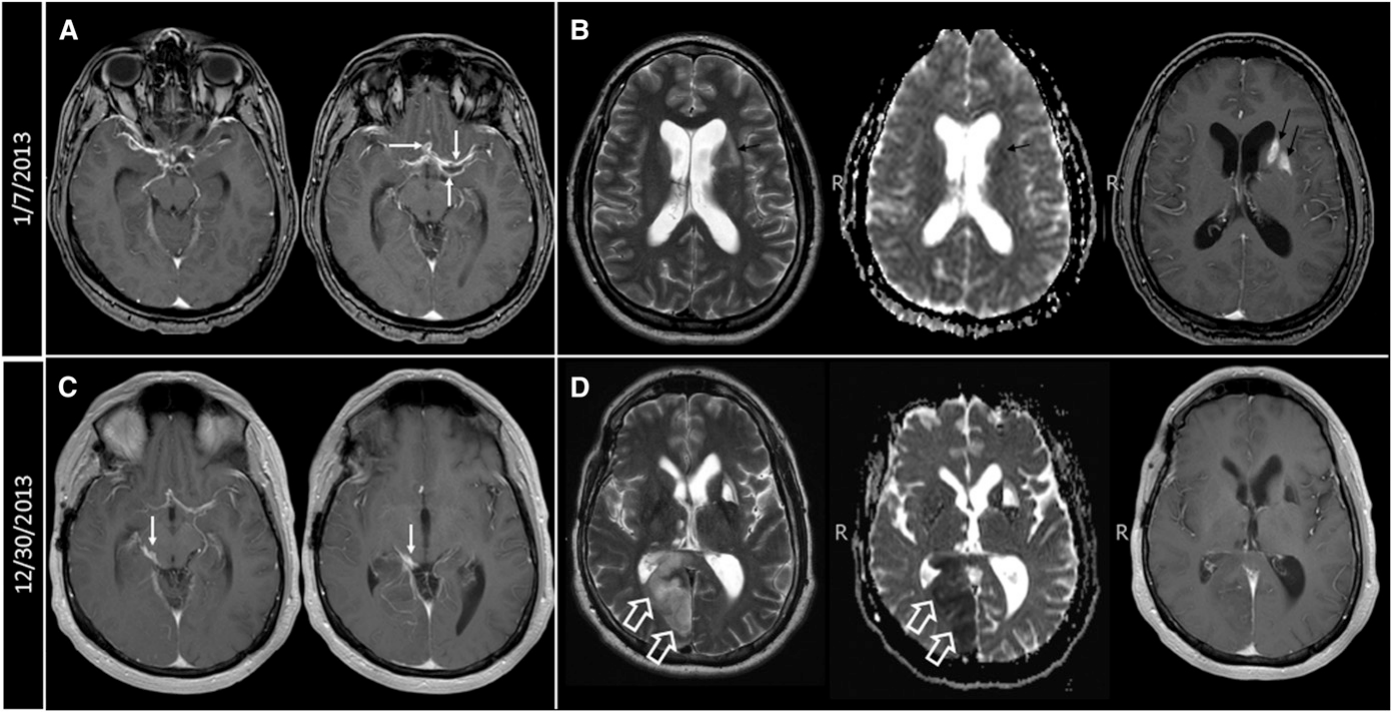
(**A**) Postcontrast T1-weighted images showing abnormal enhancement along the courses of the MCAs and ACAs bilaterally with associated cystic changes (white arrows). (**B**) Axial T2, axial apparent diffusion coefficient (ADC) maps, and axial postcontrast T1-weighted images showing subacute infarcts in the left basal ganglia and centrum semiovale (black arrows). (**C**) Abnormal enhancement in the right side of the perimesencephalic cistern (white arrows) along the course of the right posterior cerebral artery (PCA). (**D**) Axial T2, axial ADC maps, and axial postcontrast T1-weighted images showing acute infarction in the right medial occipito-parietal region (white hollow arrows).

Patient 25 was well until the age 28 when on June 27, 2000, he experienced a transient episode of right-sided weakness, numbness, aphasia, and peripheral vision loss (Figure 1). He was hospitalized but an extensive evaluation failed to reveal a definite cause. At that time, an MRI examination showed no abnormalities despite his abnormal history and highly abnormal CSF (39/mm^3^ WBCs, 68% lymphocytes, 1% eosinophils, 35 mg/dL glucose, and 57 mg/dL protein). He recovered without sequelae. About 4 years later on May 29, 2004, he experienced a similar transient episode and a repeat evaluation was unrewarding. He did well until late December 2012, when he experienced transient focal symptoms of a right-sided facial droop, fatigue, and trouble typing progressing to numbness of the fingers, hand, arm, and face; expressive aphasia; and transient confusion, prompting hospitalization on January 6, 2013. Physical and neurological examinations were normal. MRI showed abnormal meningeal/perivascular enhancement involving the Sylvian fissures, suprasellar cistern, and along the courses of the middle and anterior cerebral arteries (Figure 3A) as well as subacute lacunar infarcts involving the left basal ganglia and centrum semiovale (Figure 3B). There was medial displacement of the left posterior communicating artery by an adjacent cystic structure, which was thought to be an aneurysm but a CT arteriogram was nonconfirmatory. An LP showed CSF WBCs of 87/mm^3^ with 90% lymphocytes, glucose of 47 mg/dL, and protein of 59 mg/dL. A brain biopsy revealed cestode tegument, and a cysticercosis serology was positive for *T. solium* antibodies. The patient was born in the United States and lived in Chicago and had traveled extensively as a child but exposure was limited to day excursions to Mexico and Colombia from cruise ships in the late 1980s and one limited cross-border visit to Tijuana, Mexico. He carefully avoided ingesting potentially contaminated food and drink. The patient was started on high-dose corticosteroids and albendazole on December 31, 2012, and initially evaluated at the NIH on April 9, 2013. He complained of fatigue and difficulty with calculating and word finding, though. Physical and neurological examinations were otherwise normal. A subsequent LP at the NIH on July 30, 2013, after stopping albendazole on May 30, 2013, showed improved parameters with a WBC count of 7/mm^3^ and a borderline low cestode antigen level. An MRI showed overall decreased enhancement throughout the brain. He did well until December 13, 2013, when he complained of transient vision loss and left-sided heaviness. An LP on December 23, 2013, revealed a marked increase in CSF WBCs to 420/mm^3^ with 94% lymphocytes associated with a right posterior cerebral artery infarct (Figure 3C and D). He was restarted on albendazole, high-dose corticosteroids, and subsequently etanercept, methotrexate, and praziquantel. Repeat LPs at the NIH on February 16, 2014 and April 29, 2014, showed WBCs of 27/mm^3^ (89% lymphocytes) and 6/mm^3^ (93% lymphocytes), respectively, and no detectable cestode antigen. Cysticidal medications were stopped in July 2014 with continuation of immunosuppressive medications because of the possibility of ongoing perivascular inflammation due to nonviable degenerating cysts. After 5 years, he is clinically stable but with residual left homonymous quadrantanopia, personality changes, and difficulty with some essential cognitive functions, all contributing to a work limiting disability.

#### Illustrative case C.

**Figure 4.**
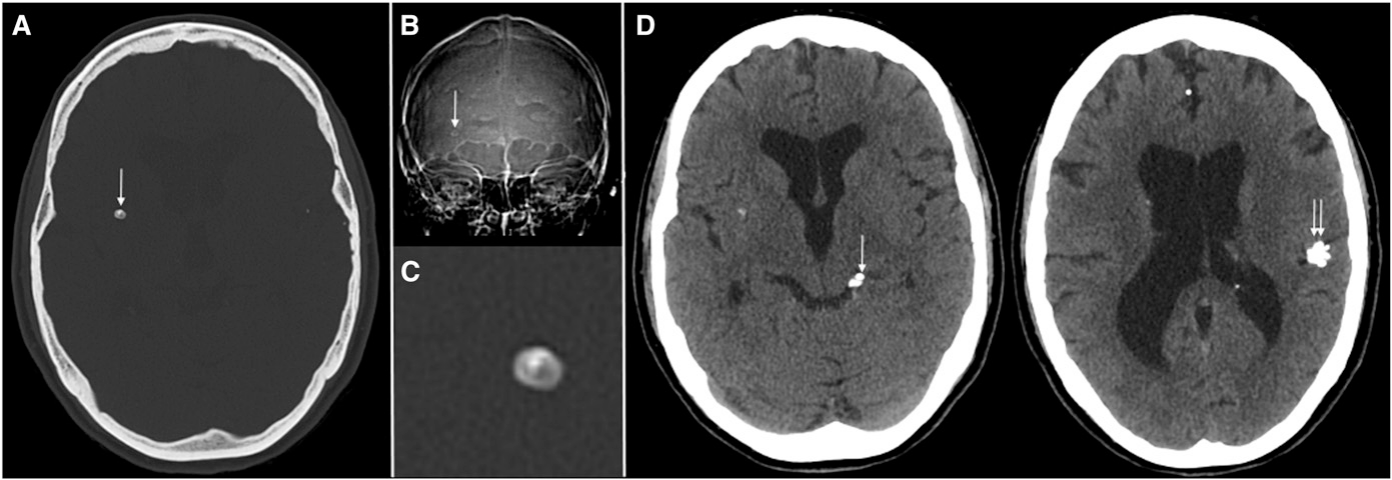
(**A**) Axial CT scan image (July 20, 2017) in bone windows, showing right basal ganglia calcific lesion with typical outline of a cyst with scolex. (**B**) Scout view of the CT scan showing the cyst with scolex (white arrow). (**C**) Magnified view of the cyst on CT. (**D**) Soft tissue windows of the brain showing multiple other calcific lesions, mainly in the left perimesencephalic cistern (white arrow) and left Sylvian fissure (double white arrows). Smaller calcifications are seen in the anterior interhemispheric fissure and in the left lateral ventricle.

Patient 34 is a 24-year-old female (Figures 1 and 4) who spent the first 4 years of life in endemic regions of South America and the Caribbean. In 1961 at the age of 4, she developed diplopia, blindness, retrobulbar headache, and a seizure in the setting of an acute febrile illness of unknown cause. The patient’s mother was told she (the mother) had an unknown infection due to ingestion of pork. In 1971 and 1976, she experienced nonmedically evaluated week-long episodes of severe retrobulbar headaches and diplopia. Evaluation of the last episode in March 1979, while an 18-year-old student in the United States, revealed papilledema, hydrocephalus, and numerous calcifications with positive NCC serology diagnostic of NCC. An LP showed 144/mm^3^ WBCs, 9% polymorphonuclear cells, 91% lymphocytes, and 41 mg/dL glucose. She was treated with a course of corticosteroids and symptoms resolved. She was evaluated at the NIH for a second opinion regarding her health status as a condition for employment on September 5, 1985. She was asymptomatic, CSF evaluation was normal, CT detected multiple parenchymal and two bulky subarachnoid calcifications, and MRI showed no viable cysts and no indication of active disease. The patient was next seen on December 5, 2017, after an episode of unconsciousness. Subsequently, she experienced a transient period of aphasia, most consistent as a focal seizure prompting resumption of anti-seizure medication. Evaluation was otherwise nondiagnostic. The patient had a prolonged untreated history of both parenchymal and subarachnoid NCC leading to compensated hydrocephalus. At least one of the episodes was accompanied by inflammatory CSF parameters, hydrocephalus, and papilledema consistent with SUBNCC. Although both degenerating ventricular and subarachnoid-located cysts can result in hydrocephalus and CSF pleocytosis, there is no evidence supporting the presence of ventricular cysts. By contrast, there are calcifications within the subarachnoid spaces consistent with prior subarachnoid cysts.

## RESULTS

### Cohort information.

Thirty-four of 134 (25.4%) patients with NCC were diagnosed with SUBNCC (Table 1). These included patients 1^19^ and 34 who had inactive SUBNCC with large calcifications within one or more cisterns. In addition, patient 1 was given a short course of albendazole and corticosteroids for treatment of a viable cyst.^19^ His symptoms, signs, treatment, and outcome were excluded from some analyses because these were not due to SUBNCC involvement. Patient 2 had a large partially calcified suprasellar mass felt to be a craniopharyngioma (Supplemental Data, medical course of selected patients). However, histopathology of the removed tissue revealed fibrous tissue enclosing a structure containing degenerated cyst remnants within the subarachnoid space. The clinical presentation and location within the subarachnoid space resembled SUBNCC even though the histopathology was not diagnostic of SUBNCC. This patient was never treated for cysticercosis and is also excluded from inappropriate analyses. Although patient 4 had severe SUBNCC, he was lost to follow up and therefore his treatment was aborted and his subsequent clinical course unknown.

**Table 1:** Demographics of racemose disease

	Median (range)
Parameter	
Total with racemose disease	34
Active disease	30
Inactive (calcified) disease	4
Male (%)	58.8
Hispanic (%)	82.4
Travel related (%)	5.9
Age parameters (years)	
At enrollment, *n* = 34	37.9 (66–25)
At diagnosis, *n* = 34	35.6 (16.4–66.2)
At initial symptoms occurred, *n* = 34	29.7 (4–64.1)
Patients who did not revisit endemic regions, *n* = 17	24.3 (7.7–46.2)
At immigration, *n* = 30	24.5 (7.7–49.3)
Time parameters (years)	
Duration from immigration to symptoms (*n* = 27)	10.0 (2–25.6)
Patients who did not revisit endemic regions *n* = 17)	10.0 (0.18–25.6)
Duration of symptoms before immigration (*n* = 4)	−5.0 (2–9.5)
Duration from immigration to diagnosis (*n* = 30)	12.8 (1–33.6)
Patients who did not revisit endemic regions (*n* = 22)	10.3 (1–27.0)
Duration followed up at the NIH	5.3 (0.2–33.2)
Duration from end of cysticidal treatment (*n* = 28)	4.2 (0.2–18.1)
Duration of disease since diagnosis, including follow-up*	8.3 (0.01–39.9)

* From diagnosis to last NIH visit.

Patients were diagnosed in their mid-30s (median of 35.6 years), mostly Hispanic, with a slight predominance of males (Table 1). They have been followed up at the NIH for a median of 5.3 years, including a median of 4.2 years after cessation of cysticidal treatment. Most patients developed symptoms a median of 10 years after immigration and were diagnosed about 2–3 years later. Seventeen patients had not revisited endemic regions and began to have symptoms after arrival in the United States. This subgroup had a minimal median incubation period of 10 years (migration to symptoms). The most likely exposure was during their residence in their endemic country. Although the exact time of exposure cannot be known, using a methodology of Dixon and Lipscomb^20^ who used the mid-point of exposure or time of residency in country (12.2 years) as the date of infection in their classic epidemiology study, we estimated the median time of incubation as 22.2 years (10 years + 12.2 years). The long incubation period is similar to the time reported in the few travelers who developed subarachnoid disease,^21^ including a long-term untreated patient in this series (patient 27) and illustrated patient B (patient 25) and a prior estimate from a review series.^12^

### Intermittent symptoms before diagnosis.

The natural history of untreated subarachnoid racemose NCC is many times punctuated by episodic transient focal neurological symptoms even years before the diagnosis is established.^22–24^ Seven of our 34 patients experienced this pattern. The reasons for failing to diagnose NCC as the cause varied among the patients. As examples, patient 25 (illustrative Case B; Figure 1, Supplemental Data, medical course of selected patients) was hospitalized twice with cryptic NCC not visualized on MRI 9 and 13 years before diagnosis. Patient 34, illustrative Case C, suffered transient episodes of headache, vision loss, and diplopia beginning at 4 years of age and was eventually diagnosed at 18 years of age. Patient 26 suffered a stroke and right-sided paralysis (subsequently diagnosed as a lacunar infarct) in Guatemala at age 24, 10 years before typical symptoms developed and the diagnosis of NCC was established (Supplemental Data, medical course of selected patients). Patient 28, who migrated to the United States from India (Supplemental Figure 4), was undiagnosed for 15 years, despite multiple clinical flares and evaluations at referral institutions/hospitals for spinal symptoms in the setting of lymphocytic pleocytosis.^25^

### Other NCC involvement.

Half of the cohort had one or more other disease manifestations of NCC (Table 2). Parenchymal calcifications, the most frequent other involvement, were present in 44.1%; the ventricular systems were involved in 20.6%. The presence of viable parenchymal cysts was uncommon, occurring in 8.8%. One explanation is that in this largely non–re-exposed population, parenchymal cysts resolved by the time racemose subarachnoid disease became symptomatic. In 70.6% of patients, multiple subarachnoid spaces were involved, whereas 29.4% had only one space involved. One or both of the Sylvian fissures were involved in 55.9% and one or more basilar cisterns were also involved in 55.9%. Four (11.8%) and three (8.8%) patients had singular involvement of the Sylvian fissures and spine, respectively, and one patient each demonstrated one lesion in the cisterna magna and suprasellar space, respectively. Severity of disease varied. Patient 13 (illustrative case 1, Figure 1)^17^ had extremely severe disease consisting of massive enlargement of almost all the cisterns and both Sylvian fissures. He was referred to us after years of suboptimal treatment that resulted in a poor clinical outcome. In two instances, cystic enlargement was not present or obvious but demonstrated diffuse arachnoiditis, suggesting other diagnoses (patient 19, Supplemental Data, medical course of selected patients, Supplemental Figure 2; patient 25, illustrative Case B, Figure 1). By contrast, a number of subjects were found to have asymptomatic subarachnoid involvement during evaluation for other diseases. For instance, patient 27 (Supplemental Data, medical course of selected patients and Supplemental Figure 3) was found to have an ambient cistern cyst during evaluation for a pituitary adenoma. An MRI obtained in patient 31 (Supplemental Data, medical course of selected patients) for evaluation of worksite trauma detected asymptomatic right Sylvian fissure involvement as well as an asymptomatic 4^th^ ventricular cyst. Patient 10 (Supplemental Data, medical course of selected patients) presented in extremis with 3^rd^ ventricular cyst and was found to have an asymptomatic right Sylvian fissure cyst and subarachnoid involvement of the suprasellar and left ambient cisterns.

**Table 2:** Other types of NCC involvement

Types of NCC	Number/total (%)
S only (percent)	17/34 (50)
S, C	8/34 (23.5)
S, C, P	1/34 (2.9)
S, C, P, PE	1/34 (2.9)
S, C, V	4/34 (11.8)
S, C, V, P	1/34 (2.9)
S, V	2/34 (5.9)
SP involvement	12/34 (35.3)
SP as the only involvement	3/34 (8.8)
Total C (%)	44.1%
Total PE	2.9%
Total P	8.8%
Total V	20.6%

C = calcification; NCC = neurocysticercosis; P = viable parenchymal; PE = perilesional edema; S = subarachnoid; SP = spine; V = ventricular.

### Disease manifestation and complications.

The complications due to NCC in this series are similar to prior reports (Table 3).^10,12^ Increasing frequency and severity of headaches commonly led patients to seek medical advice. Among the patients, 55.9% developed hydrocephalus (and associated symptoms) with 41.2% requiring a VP shunt and one person undergoing ventriculostomy of the 3^rd^ ventricle (patient 21). Spinal involvement occurred relatively frequently, in 35.3% of our cohort and three of these 12 as the only involvement.^26^ Pain from lumbar SUBNCC occurred in 14.7% and in five was the most prominent morbidity. One or more infarcts, arguably the most serious complication in SUBNCC, were detected on MRI in six (17.6%) patients on admission. One of these (illustrative Case B, patient 25, Figure 1) developed a major vessel infarct while under observation for several months posttreatment and was the only person to experience a major vessel infarct or any infarct while being followed up. Multiple lacunar infarcts caused most of the symptoms and morbidity in two patients with diffuse arachnoiditis (patient 25, illustrative Case B, Figure 1) and patient 19 (Supplemental Figure 2 and Supplemental data: medical course of selected patients). Patients with this manifestation may be particularly susceptible to infarcts.

**Table 3:** Complications

Complication	Number (%)
Hydrocephalus	19/34 (55.9)
Shunt	14/34 (41.2)
Ventriculostomy	1/34 (2.9)
Spinal pain symptoms	5/34 (14.7)
Infarct (%, median, range)	6/34 (17.6, 3, 1–6)

Large vessel involvement was uncommon. Asymptomatic calcifications involving the middle cerebral arteries along their course in the Sylvian fissures were seen in two patients, which is likely a result of inflammation directed toward prior degenerating Sylvian fissure cysts. Patient 17 (Supplemental Figure 2 and Supplemental Data: medical course of selected patients) developed an asymptomatic inoperable basilar artery aneurysm that remained unchanged during and after treatment. Patient 25 (illustrative Case 2, Figure 1) suffered a large vessel stroke, which may have been due to persisting inflammation from nonviable parasites.

### Concomitant diseases.

Patient 8 and 33 in addition to biopsy proved that SUBNCC had other brain diseases, which are detailed in the Supplemental Data: medical course of selected patients, Supplemental Figure 5.

### Treatment.

Drug administration information is summarized in Table 4. All treated patients (30/34, 88.2%) were given one or more courses of albendazole and all but one could be adequately assessed after treatment. Praziquantel was less frequently used (61.8%) either as a replacement or in combination with albendazole. Corticosteroids were used in all treated patients (88.2%), but efforts were made to limit their use by addition or substitution with other immunosuppressive drugs. Methotrexate^16^ and etanercept^17^ were used in 50% and 34.2% of all cases, respectively. Even though the corticosteroid taper was slow, worsening symptoms and signs commonly occurred, prompting the addition of methotrexate and/or etanercept. These drugs appeared to allow continuation of taper and control of developed symptoms/signs.^16,17^ Treatments were prolonged for albendazole (median 0.55 year, range 0.05–4.7 years), praziquantel (median 0.96 year, range 0.01–4.3 years), and for the immunosuppressive medications, including corticosteroids (median 1.12 years, 0.02–5.0 years), methotrexate (median 1.37 years, range 0.08–7.92 years), and etanercept (median 0.81 year, range, 0.11–1.91 years). Anakinra, an interleukin 1 receptor antagonist, was prescribed only in patient 28 before the diagnosis of NCC and continued after the diagnosis in an attempt to better control spinal nerve pain.^25^ Consideration to stop therapy occurred when MRI imaging was improved with moderation and stabilization of enhancement, decreased or serial decreases in cestode antigen concentrations to low or undetectable levels, successive decrease in CSF WBC counts, and improved or stable clinical state of the patient.

**Table 4:** Treatments and duration

Treatment (range)	Number (%)	Median duration of all treatments in years (range)
Albendazole*	30/34 (88.2)	0.55 (0.05–4.7)
Praziquantel*	20/34 (61.8)	0.96 (0.01–4.3)
Combined*	20/34 (61.8)	NA
Corticosteroids*	30/34 (88.2)	1.09 (0.02–5.0)
Methotrexate	17/34 (50.0)	1.37 (0.08–7.92)
Etanercept*	11/34 (34.2)	0.81 (0.11–1.91)
Anakinra	1/30 (0.03)	1.61

NA = not applicable.

* Three persons with calcified subarachnoid did not receive treatment and one person (who received aborted inadequate treatment cysticidal and etanercept) was excluded.

### Utility of CSF cestode antigen levels.

Serial LPs to evaluate changes in CSF parameters and cestode antigen levels before, during, and after the treatment were useful to determine effectiveness of the treatment, degree of brain inflammation, and the presence of recurrent infection/disease. In patients in whom cystic components were not present or who had responded to therapy and had unchanged MRIs over time, it was a good measure of efficacy and continued inactive disease and sometimes the only way to guide treatment and to detect disease recurrence. Change in lumbar CSF WBC counts and cestode antigen levels over time in relationship to treatments are shown in 15 treated patients in whom serial CSF samples are available, including one CSF at the end or after treatment (Figure 1, Supplemental Figures 1–5). Table 5 shows disease status according to number and timing of CSF evaluations. Cestode CSF antigen levels that fell to undetectable levels, usually accompanied by improved WBC counts, predicted sustained inactive disease. Of the 17 courses (16 patients) with serial measurements near or after the end of the treatment, 13 had no detectable antigen and all had inactive disease, although three have not yet reached a year after the end of the treatment. Two had borderline levels resulting in an asymptomatic recurrence in one and sustained inactive disease in the other. The remaining patient with high antigen levels continues to have active disease. Of the eight patients (eight courses) with a single CSF value, three had posttreatment CSF cestode antigen measurements, two showed no detectable cestode antigen, and one had an elevated level, but CSF was obtained after a recent extraction of a cisterna magna cyst. Five had CSF values determined significantly before the end of the treatment. All have sustained the inactive disease. Except for the one patient lost to follow-up, the remaining seven treated patients without CSF samples have sustained the inactive disease status, likely because of the effectiveness of long-term treatment. Reaching no detectable antigen in the CSF was highly predictive of sustained inactive disease (*P* ≤ 0.01, Fisher’s exact test).

**Table 5:** Disease activity related to knowledge of CSF cestode antigen status level

CSF status		Inactive	Active	Not assessable**
Serial lumbar CSF values*	17			
No detectable antigen	13	10	0	3
Borderline-low antigen	2	1	1	0
Elevated antigen	1	1		
High antigen^	1	0	1	0
Single lumbar CSF value	8			
No detectable antigen post treatment	2	2	0	0
Elevated but obtained before or during treatment^#^	5	5	0	0
Elevated post treatment^$^	1	1	0	0
Untreated, calcified lesions^%^	2	3	0	0
No CSF obtained	7	7	0	0
Lost to follow up	1	0	0	1
Total evaluable (%)*	35	29 (82.9)	2 (7.2)	4 (11.4)
Treated patients, not lost to follow-up	30	26 (86.7)	1 (3.2)	3 (10.0)

* Last lumbar CSF cestode antigen level just before or after stopping cysticidal treatment in all but the patient with high antigen^ who has ongoing active disease. Includes 2 assessments in one patient who relapsed and was then successfully retreated. Includes one patient with calcified suprasellar lesion, positive antigen that reverted to negative after removal.

** Less than year follow-up post treatment.

^ Uncured, asymptomatic, MRI stable without obvious involvement despite years of treatment, now on combined high dose albendazole and praziquantel without immunosuppressive medications.

# Positive antigen too early to be predictive or interpretable. One patient had an undefined non tumor proliferative lesions in the spine and sella.

$ Cisterna magna cyst removed 5 weeks earlier. Untreated.

% One patient with a calcified suprasellar lesion had positive antigen that became negative after removal. A second patient had negative antigen and a third patient was treated about 2 weeks for an accompanying viable parenchymal lesion and no CSF was obtained.

*** Comparison of the disease activity at the end of treatment in relation to the presence of any antigen in the CSF at or after treatment showed that lack of antigen predicted inactive disease, *P* ≤ 0.01 (Fisher’s exact test).

### Recurrent infection/disease.

Recurrent infection occurred in three patients. The CSF cestode antigen value of patient 9 (Supplemental Data: medical course of selected patients, Supplemental Figure 1) fell to a nearly undetectable level. However, 2.7 years following cessation of cysticidal treatment infection recurred. Two other patients, who lacked pretreatment cestode antigen levels had recurrent infection/disease. Patient 19 (Supplemental Data: medical course of selected patients, Supplemental Figure 2) experienced an asymptomatic recurrence associated with an elevated CSF cestode antigen level and an increased CSF WBCs that became normal and undetectable in response to retreatment. Patient 8 (Supplemental Data: medical course of selected patients) suffered a symptomatic relapse of spinal disease accompanied by the growth of a new lumbar cyst, elevated cestode antigen, and mildly elevated CSF WBCs. Conversely, serial elevated cestode antigen values indicated continued active disease. Patient 20 (Supplemental Data: medical course of selected patients, Supplemental Figure 7) has been treated repeatedly over 12 years but still has active disease. After cysticidal treatment, all treatments were stopped and subsequently cysts regrew.

### Other changes in CSF parameters.

Serial WBC counts in the CSF (all lymphocytic predominant) decreased in most patients and reached < 7 WBCs/mm^3^ in 6/15 patients (40.0%) near or after the end of the treatment. The median of all 15 patients was 8.5 WBCs/mm^3^ (range 1–54 WBCs). In two patients, low to moderate elevated counts (up to 54/mm^3^) persisted for years post cessation of therapy in the absence of recurrent infection/disease or rise in cestode antigen. No patient showed a neutrophilic predominance while enrolled at the NIH, although one patient with a single CSF analysis among many that showed neutrophilic predominance before NIH enrollment. In 5/17 patients (29.4%), at least one CSF in the prior records or at the NIH showed greater than 5% eosinophils; however, this is likely a low estimate because eosinophils were not consistently reported or quantified in the CSF WBC differential. Hypoglycorrhachia (< 46 mg/dL) occurred one or more times in 68% (15/22) of patients and tended to resolve over time as the patients’ clinical status improved. However, increases were not as consistent as a biomarker of effective therapy compared with a decrease in CSF WBC numbers or decline and loss of CSF cestode antigen.

### Long-term clinical outcome.

The clinical status a median of 4.2 years after treatment cessation is summarized in Table 6. In contrast to earlier reports and general reviews associating high morbidity and mortality with subarachnoid racemose disease,^10,27–29^ the long-term outlook in this series is much better. There were no deaths or severe disability that required institutionalization. All patients were able to care for themselves with the exception of patient 25 who sustained a large vessel stroke (illustrated Patient 2, Figure 1) and patient 13^17^ (Supplemental Figure 2) who can now work and drive but needs family assistance for decision-making. Patient 33 (Supplemental Data: medical course of selected patients, Supplemental Figure 5) who had a non-NCC suprasellar mass became legally blind as a result of the undefined process unrelated to NCC involvement. In most patients, neurological symptoms substantially improved concomitant with initiation of high-dose corticosteroids, but they then developed symptoms related to high-dose corticosteroids to varying degrees such as diabetes in 41.1% (14/34, most resolved after corticosteroids were stopped), and aseptic necrosis of one or more joints in 24.2% (8/33). Although 42.4% (14/33) had no complaints and fully recovered, 24.2% (8/33) were disabled and did not work; 24.2% (8/33) suffered from episodic neurological symptoms, including seizures and perilesional edema from parenchymal disease, and transient episodes of focal neurological symptoms, including hemiparesis and aphasia from subarachnoid disease by unclear mechanism; and 15.2% developed mild intellectual impairment.

**Table 6:** Posttreatment status

Status	Number/total (%)
Well without sequelae	14/33 (42.4)
Lost to follow-up	1/34 (2.9)
Unresolved infection	1/33 (3.0)
Residual hydrocephalus	2/33 (6.0)
Mild intellectual impairment	5/33 (15.2)
Unable to work*	8/33 (24.2)
Visual impairment	3/33 (9.1)
Episodic neurological symptoms	8/33 (24.2)
Neurological sequelae from stroke	2/33 (6.0)
Depression	4/33 (12.1)
Seizures	2/33 (6.0)
Headaches	2/33 (6.0)
Poor balance/walking	3/33 (9.1)
Focal neurological weakness	4/33 (12.1)
Aseptic necrosis	8/33 (24.2)
Secondary CNS pathology	2/33 (6.0)
Basilar artery aneurysm	1/33 (3.0)

* Patients’ assessment.

## DISCUSSION

Treatment approaches to SUBNCC vary because the pathophysiology and natural history of disease are imperfectly understood and there are no rigorous randomized treatment trials to guide therapy. Definition of treatment success and recurrent infection/disease are lacking and there are no acceptable predictive tests to follow up treatment or to define when to stop the therapy.

Some investigators use a short 8–10-day course of high-dose albendazole and dexamethasone or 15 days of praziquantel^28^ with cyst regression as a measure of effectiveness.^10,28,30–33^ However, this regimen is frequently unsuccessful, requiring one or more retreatments.^33,34^ Furthermore, the duration of follow-up is often short and longer term outcomes, including morbidity, mortality, side effects of treatment, and recurrence rates of this treatment approach, are unclear. Proaño et al.^35^ treated patients with one to three giant subarachnoid cysts causing mass effects (mostly Sylvian fissure located cysts) with high-dose corticosteroids and one or more 4-week-long courses of albendazole or praziquantel. Response was assessed by cyst size change; 63.7% had recurrent or continuing disease and required more than one courses of cysticidal treatment. These patients were followed up a median of 4.9 years, although the details of follow-up were not described. This study reinforces the need for protracted cysticidal treatment and adequate control of inflammation. We used prolonged high-dose cysticidal and immunosuppressive therapy for a sufficiently long period to allow improvement in a number of treatment-effected parameters. The duration of treatments and dosing were guided by combined measures of clinical improvement, MRI-determined imaging changes, including cyst degeneration and level of enhancement, normalization of CSF parameters, and undetectable CSF cestode antigen concentration. After cessation of the treatment, the patients were closely monitored and then minimally every year for as long as possible. This approach led to much-improved clinical outcomes of no deaths, moderate morbidity, and three recurrences (9%) after a long-term median posttreatment follow-up of 4.2 years.

The rationale for prolonged cysticidal and anti-inflammatory treatment is based on the unique biology of this form of *T. solium*. In contrast to normal structured cysts that are capable of developing into a tapeworm, SUBNCC consists of structurally aberrant slowly proliferating *T. solium* larva that most commonly invade into and expand within the cisterns surrounding the brain.^3–5^ A degenerated scolex is sometimes present but characteristically absent. The original description by Virchow^36^ and more fully characterized by Zenker^8^ was primarily gross descriptions of the multivesicular network of vesicular structures that Zenker called “*Cysticercus racemosus*” of racemose NCC. However, a more accurate description of subarachnoid NCC taken from more current pathological descriptions,^2,6,37^ MRI imaging, and response to treatment should include vesicular (e.g., racemose) and nonvesicular proliferative diseases. Vesicular structures may not have formed, be present microscopically, or have earlier degenerated and lost their fluid interior as a result of naturally occurring host inflammatory responses or as a consequence of treatment. Combinations of viable and degenerated parasite tegument along with variable degrees of inflammation are well described in SUBNCC.^2,6,37^ Whether or not gross multicystic vesicles are present or just parasite tegument, disease is caused by inflammation directed to the parasite that has invaded the subarachnoid spaces of the brain. We clearly document the growth of typical racemose cysts from residual, viable, nonvesicular parasites, indicating the disease is due to same pathophysiology but different growth forms. Whether the term “racemose NCC” should be applied to this type of disease seems reasonable but, in this context, would not be limited to a description of multivesicular disease.

Reports in the literature^34,38^ and case histories reported here clearly demonstrate that short-term treatments are frequently only partially effective leading to recurrent infection/disease. Prolonged treatments and long-term follow-up are required to maximize cure rates and to detect treatment failures. Potential reasons for recurrences include parasite’s bulk and large size, inherent difficulty in killing *T. solium*–proliferating cells, the ability of metabolically hypoactive or slowly dividing cells to survive, or suboptimal CSF drug levels. Inflammation, the major cause of serious sequelae, is usually limited in mostly cystic SUBNCC^5,6,13^ but dependably develops and worsens with parasite degeneration. Because the parasite is large and macroscopic, resulting in a massive parasite burden, and is frequently widespread, the ensuing inflammation is extensive, chronic, and damaging. Therefore, control of inflammation is critical to avoid serious sequelae.^39,40^

From these cases, a clearer understanding of the natural course of subarachnoid disease can be gleaned. Illustrated Case A, patient 7, presented with marked cystic disease involving multiple cisterns and resembles the original description of racemose from of SUBNCC. At this stage (Stage 1), the cysts cause mass effects and the presence of inflammatory responses is limited or does not yet dominate.^5,6,13^ Although this particular patient returned to an endemic region yearly since immigration some 20 years earlier, data from our other patients well document and confirm other reports that subarachnoid disease usually becomes clinically evident after a prolonged incubation period.^12,21^ We found a minimal median incubation period of 10 years and an estimated median incubation period of more than 20 years, indicating that in most cases, the parasite grows slowly. From this series and other case reports, it is evident that symptoms eventually occur from mass effects and/or ensuing inflammation. Treatment or host recognition initiate or worsen the inflammatory response transforming fluid-filled cysts into collapsed structures outlined by enhancing host-derived arachnoid and/or a diffuse inflammatory response to parasite antigens.

Patient 25, illustrated Case B, is characterized by pronounced inflammation (Stage 2) that had been causing symptoms and signs for at least 13 years before diagnosis. Although limited cystic structures were identified at diagnosis, they were not seen earlier, were few in number, and went largely unrecognized. Manifestations were mostly due to inflammatory reaction to noncystic parasite tissue and tegument in the subarachnoid spaces resulting in vasculitis and lacunar infarcts.^6,22,41–44^ Most patients demonstrate a combination of cystic elements and inflammation, with the latter predominating in chronic disease resulting in most of the serious manifestations. This patient had other unusual aspects regarding the infection. He had minimal exposure history, a prolonged incubation period, and long-standing, cryptic disease 13 and 9 years before diagnosis. Even though there were significant symptoms and signs at those times, including an inflammatory CSF profile, the MRIs were surprisingly unrevealing (13 and 9 years before diagnosis).

Illustrative Case C, patient 34, is representative of end-stage inactive disease (Stage 3) without viable parasites or significant inflammation, which occurs naturally as in this patient or as a result of treatment. Long-term follow-up of our treated patients revealed gradual resolution of disease with residual scarring, atrophy of adjacent brain, diminution of walled-off cysts defined by enhancing arachnoid membranes, residual arachnoid cysts, and subarachnoid calcifications at the site of prior degenerating parasites. Prior disease may be hardly discernible. In this patient, resolution without serious sequelae occurred as part of the natural progression of untreated SUBNCC. Most of her course was not documented, but it is likely her symptoms were due to degenerating SUBNCC because there were large calcifications within two subarachnoid cisterns and there was no evidence of ventricular involvement, which could cause a similar clinical course. The initial evaluation at the NIH revealed multiple asymptomatic parenchymal calcifications and inactive subarachnoid disease that resulted in compensated hydrocephalus. About 33 years later, she again came to our attention for evaluation of a likely seizure disorder probably caused by parenchymal cysticercosis. A 30-year incubation period to develop epilepsy was previously documented.^20^ In the present case, epilepsy developed about 52 years likely after the infection.

The importance and morbid and mortal consequences of inflammation in this disease has been known for over a century,^3^ but the value, use, and duration of anti-inflammatory treatments in SUBNCC to effectively control inflammation and prevent sequelae is unproven and hardly studied.^16,17,39^ Nevertheless, despite lack of definitive information, corticosteroids are almost universally recommended. Because inflammation in SUBNCC is substantial, chronic, injurious, and increased by cysticidal treatment, we used long-term immunosuppressive treatment. Quick reversal of symptoms is common after the start of high-dose corticosteroids, but significant side effects commonly ensue. Most can be treated or are lessened and resolve after lowering and stopping the treatment. To decrease and avoid toxicities of corticosteroids, we used and reported the use of methotrexate^16^ and etanercept^17^ for corticosteroid sparing and replacement. Anecdotal reports suggest efficacy, but randomized trials are needed. In addition, use of combined albendazole and praziquantel treatment has been shown to be safe and more effective than albendazole alone in multicystic parenchymal disease,^45^ and although not prescribed to all patients, could have contributed to more effective treatment and good clinical outcome.

The use of biomarkers to monitor treatment has been suggested previously but their utility unproven.^46–50^ Our results substantially suggest their value. Quantitative cestode antigen levels in the CSF tended to decrease with treatment and an undetectable level near or after treatment ceased predicted sustained disease inactivity. This is a logical biomarker to follow because this antigen is secreted by viable *T. solium* cysts and inhibited with cysticidal treatment.^51^ Serial CSF evaluations were also helpful to validate treatment effectiveness. The number of CSF WBCs also decreased with treatment over time, but a majority did not reach normal values at the end of the treatment. Drug-induced cyst degeneration is a treatment effect, but an imperfect indicator of sustained disease inactivity. Other patients lack cystic components and yet have ongoing disease. Our approach is to follow up complicated cases with serial posttreatment LPs and annual to semiannual MRIs. No relapses have occurred with patients reaching two negative cestode antigen values 2–3 months apart. Although results are not shown, we did not find plasma cestode antigen levels as sensitive as CSF antigen levels and following only blood value does not provide information regarding the presence and degree of arachnoiditis.

There are important caveats regarding these results. The treatment used is prolonged, expensive, and causes significant side effects mostly due to long-term high-dose corticosteroids, including diabetes, Cushing’s syndrome, and aseptic necrosis of joints. Use of corticosteroid-sparing/replacement agents is also expensive and their benefits are suggestive but unproven. Our patients had free care, including medications, and were followed up intensively by an experienced and knowledgeable team, likely contributing to the excellent clinical outcome. In less supportive environments, outcomes may not be as good. Although the study indicates the effectiveness of prolonged treatment, shorter treatment regimens were not studied and need to be evaluated.

## Supplemental data and figures

Supplemental materials
