# Supplementary material for: Natural History of Treated Subarachnoid Neurocysticercosis
Source: Am J Trop Med Hyg. 2019 Oct 21;102(1):78–89. doi: 10.4269/ajtmh.19-0436 (PMC6947806; doi:10.4269/ajtmh.19-0436)
Supplement: Supplementary file 1 [file tpmd190436.SD1.pdf]

Supplemental Data: Medical Course of Selected Patients

Patient 2

Paitient 2 is a 51-year-old female who immigrated from Nicaragua to the U.S in 2009. She related a 5-year history of worsening headaches culminating in 2 documented emergency room visits for headaches, nausea, vomiting, blurry vision, and loss of balance. MRI in November of 2010 showed a suprasellar and right pituitary mass that was most consistent with craniopharyngioma. She was first seen at NIH in January of 2011 and the mass was subsequently removed in March of 2011. Histopathology revealed a recognizable degenerated cestode including calcareous corpuscles surrounded by host derived fibrous tissue. CT examination after surgery showed a small calcification in the right parietal lobe consistent with NCC but no other NCC lesions. Shortly after surgery a lumbar CSF cestode antigen was abnormally increased, which became undetectable 5 months later in the absence of additional treatment. Histopathology of the removed mass showed recognizable cestode structures but contained remnants of the scolex without proliferating membranes typical of racemose parasites. However, the presentation and presence in the subarachnoid space mimicked racemose cyst and is therefore is included.

Patient 8

Patient 8 is a 27 y/o female who had extensive symptomatic spinal NCC, surgical extraction, and long-term medical treatment prior to her initial NIH evaluation on 2/10/09. The patient immigrated to the U.S. from El Salvador at about 7-8 years of age in 1989 or 1990. In 2007 she developed numbness and weakness starting with the lower left leg progressing over the ensuing 6 months to involve both legs, the lower back with urinary retention and incontinence. In December 2007 an MRI revealed extensive NCC cystic disease from T12 to L5 and a possible sacral cyst as well. Surgery in December of 2007 evacuated at least 22 cysts, but not all the cysts could be extracted. No imaging was available for review before surgery and subsequent imaging did not reveal brain involvement. At presentation to NIH on 2/10/09 she was asymptomatic. Cysts previously identified post operatively at C6-7, T2, and three between L1-5 had resolved on low dose corticosteroid and albendazole that she had been taking for over a year. All drugs were therefore stopped. On 6/30/10 the patient complained of a 2-week history of right leg pain radiating from the buttock to the thigh and posterior aspect of her right calf associated with decrease in touch sensation and loss of ankle reflex. MRI showed a new cyst at L5. CSF, obtained with difficulty on 7/14/10, showed 12 WBC/mm^3, 81% lymphocytes, glucose 44 mg/dl, protein 35 mg/dl and an elevated cestode antigen level. Albendazole, praziquantel, dexamethasone and cimetidine were started on 7/28/10. The leg pain and cystic lesion resolved. Albendazole was stopped and replaced with praziquantel on 8/12/10 because of liver

function test (LFT) enzyme abnormalities. Prednisone and praziquantel treatment ended at the end on December 2011. Although the patient has done well without recurrence, follow up brain MRIs revealed a slowly growing solid lesion close to the right anterior ventricle, not compatible with NCC. The mass was excised but nature of the tumor could not be definitely determined.

Patient 9

The patient is 41-year-old female from Honduras who migrated to the U.S. approximately 8 years prior to presentation. About 8 months before diagnosis in July of 2012, she was evaluated for a 5-day history of numbness over half her body and headaches. She was told she had cysts in her brain but was discharged without therapy or a final diagnosis. Three months prior to diagnosis she developed worsening headaches, low-grade fever for a month and then nausea, vomiting, and dizziness, which prompted emergent evaluation on 2/9/13. MRI demonstrated hydrocephalus, leptomeningeal enhancement, and lobulated cystic masses at the base of the medulla bilaterally, which were responsible for long tract signs and symptoms. CSF showed 447 and 482 WBCs with 69% and 67% eosinophils on two separate occasions. She was started on albendazole and 30 mg prednisone daily and referred to NIH on 2/28/13. Dexamethasone 8 mg/day, praziquantel and etanercept 25mg/week were added and INH begun for latent TB. She initially responded to therapy and was discharged on 3/8/13, but on 3/11/13 she was readmitted after developing left-sided body and face numbness, blurry vision, nausea and vomiting. Dexamethasone was increased to 16 mg/day with improvement of neurological

symptoms but resulted in increased anxiety and emotional lability. Methotrexate 20 mg/week was added to her regimen and dexamethasone was decreased to 12 mg daily before discharge. On a gradual taper she developed hydrocephalus accompanied by nausea and vomiting, requiring placement of a ventricular peritoneal shunt on 4/11/13. She was again readmitted on 4/24/13 for headaches, subjective fevers and right lower quadrant pain. Her regimen was simplified to include only albendazole, methotrexate and corticosteroids, which were tapered. She did well with cyst resolution and MRI stabilization. CSF parameters cestode antigen dramatically decreased to a borderline but positive value, but CSF WBCs, although decreased, remained elevated at 28/mm^3. Albendazole, corticosteroids and methotrexate were stopped on 8/1/13, 1/1/14 and 8/1/14, respectively. The patient was briefly lost to follow up. On reevaluation on 1/20/16, the patient was asymptomatic but SUBNCC had recurred as determined by cyst regrowth, a large increase in the lumbar CSF cestode antigen level, and a marked rise in CSF WBC numbers (Supplemental figure 5). Intervening shunt malfunction and hydrocephalus necessitating shunt replacement, delayed restart of treatment. On 3/28/16 she was started on combined praziquantel and albendazole therapy and low dose corticosteroids which were quickly tapered off. Recurrent cysts degenerated and resolved, cestode antigen became undetectable and CSF WBC count fell to 5 from 332 WBC/mm^3. All treatments were stopped in late October 2016 and the patient has remained free of disease to the present.

Patient 10

The patient is a 33-year-old male who presented in extremis due to an obstructing 3rd ventricle cyst and during evaluation was found to have clinically silent SUBNCC. He migrated to the U.S. in 1994, about 16 years prior to presentation, but has returned several times to his native country Mexico. He had a 10-year history of headaches and occasional episodes of confusion. On 1/2/2010 he had a syncopal episode, altered mental status, headaches, nausea and vomiting. Evaluation revealed a large 3rd ventricular cyst obstructing CSF flow that could not be extracted. It was fenestrated on 1/8/10 and an external ventricular drain (EVD) placed. He had a prolonged hospital stay and shortly after surgery he was given a brief 7-day course of albendazole and corticosteroids of unclear dose. In addition, the patient had significant but asymptomatic subarachnoid cystic involvement of the right Sylvian fissure, suprasellar, prepontine and perimesencephalic spaces on the right and abnormal enhancement in the left perimesencephalic space indicating prior subarachnoid cystic involvement. He was first seen at NIH on 6/8/10, complained of mild headaches and declined treatment. A LP on 6/24/10 showed 44 WBC, 70% lymph, 2 neutrophils, other 28% other cells, protein 47 mg/dl and glucose 36 mg/dl. The cestode antigen concentration was significantly elevated before treatment was initiated. On 12/16/10 he was started on 10 mg dexamethasone and a lower than normal dose of albendazole, 600 mg/day, because of liver function test abnormalities and a current history of excessive alcohol ingestion. The lesions regressed and albendazole and corticosteroids were stopped around 6/23/11. A LP on 6/24/10 showed 44 WBC, 70% lymph, 2 neutrophils, other 28% other cells, protein 47 mg/dl and glucose 36 mg/dl. The cestode antigen concentration was significantly elevated at the time treatment stopped; no addition CSF was obtained. Nevertheless, he has done well without recurrence over the ensuing 8 years.

Patient 17

The patient is a 30-year-old Hispanic male who immigrated from El Salvador in 2006 and was referred to the NIH on 8/4/14 for treatment of SUBNCC. The details of treatment prior to 2014 are sketchy. He was apparently well until 2008 when he developed severe headaches and later in 2009 suffered a bout of loss of consciousness. MRI showed extensive SUBNCC including involvement of the left perimesencephalic cistern, the left Sylvian fissure and an interhemispheric cyst. He was diagnosed with NCC at that time and given a short 2-week course of medication for a “pig parasite,” likely albendazole, and corticosteroids with clinical improvement. Similar symptoms reoccurred in 2012. The prior cystic lesions had expanded with extension into suprasellar and interpeduncular spaces and a lacunar infarct in the left para-temporal region was present. He was treated with a two-week course of albendazole and dexamethasone, again resulting in clinical improvement. Following return of severe headaches in May 2014 associated with problems walking and talking, reevaluation documented hydrocephalus and SUBNCC with further extension and a newly recognized non-operable basilar artery aneurysm. The left Sylvian fissure cyst had enlarged and expanded, and another large cyst expanded laterally from the left perimesencephalic space. He was treated with a months-long course of albendazole and praziquantel and high dose corticosteroids with clinical

improvement. Hydrocephalus had partially resolved with treatment. On referral to NIH on 8/4/14 he complained of blurry vision, dizziness and headaches since stopping treatment. Neurological examination was normal. There was general improvement with residual cysts, multiple parenchymal calcified granulomas and a calcified cyst within the left perimesencephalic cistern. On 8/7/14 he was started on albendazole, dexamethasone 8 mg/day and praziquantel and methotrexate. Etanercept 25 mg/week was started on 10/23/14 to control increased enhancement and worsening hydrocephalus despite 4 mg/day of dexamethasone. The corticosteroid taper was continued but he developed new headaches and left arm numbness on 9 mg prednisone/day that resolved after a transient increase in corticosteroid dose. He tapered off corticosteroids on 7/2/15 and albendazole, praziquantel and etanercept were stopped about 3 weeks later. Initial LP and CSF analyses showed a WBC of 135/mm^3 and positive cestode antigen. The abnormal CSF WBCs along with sub-optimal decrease in size of the left temporal area and left Sylvian fissure cyst suggested continuing active disease, which prompted retreatment with combined albendazole and praziquantel and low dose corticosteroids from November 2015 until early March 2016. Serial CSF analyses initially improved, but there was an unexplained spike. Eventually, the cestode antigen levels became repeatedly undetectable suggesting inactive disease. The basilar artery aneurysm remained unchanged. The previously enlarged cysts have decreased in size but have not completely regressed. The undetectable CSF antigen levels and unchanging MRI suggest inactive disease, but CSF WBC continues to be elevated at 54 WBC/mm^3. He remains asymptomatic with unchanged serial MRIs to the present.

Patient 19

The patient is a 30-year-old Guatemalan male with meningeal neurocysticercosis resulting in multiple neurological complications. The patient has been in the United States since age 17 except for occasional trips to Guatemala, last in 1994. He was well until October 2001 when he developed intermittent dizziness with a sensation of whirling and transient spells of aphasia. These symptoms gradually improved but he then experienced bilateral dysesthesias of the legs that progressed to weakness, right (R)> left (L), and about the same time in November 2001 developed headaches, neck pain, vomiting, and double vision. An initial evaluation in December 2001 including MRIs of the brain and spine and an EMG were unrevealing. He improved almost to normal but then developed numbness and tingling of this left arm. A repeat examination led to the working diagnosis of Guillain Barré. He again improved somewhat with physical therapy but in May 2002 developed pain in the left arm, perioral numbness, double vision, dizziness, headaches and intermittent vomiting. Evaluation revealed diffuse leptomeningeal enhancement of the base of the brain extending to the suprasellar cistern, cavernous sinuses and encasement of the optic nerves and chiasm. Two cysts were noted above the suprasellar cistern and mild hydrocephalus was present. An LP showed an opening pressure > 55 mmH2O, glucose 24 mg/dl, protein 107 mg/dl, WBC 111/mm^3 with 81%

lymphocytes, 10% monocytes, 7% eosinophils, 1% basophils, and 1% neutrophils. A cysticercosis serology was strongly positive. Before treatment was initiated, he suffered a 3-hour period of confusion and disorientation and subsequently in September 2002 started on prednisone and an 8-day course of albendazole. After stopping albendazole while on 40 mg prednisone daily, he experienced 2 periods of confusion and disorientation prompting an increase of prednisone to 60 mg/day. MRI on 10/2/2002 showed improvement and decrease in size of the 2 suprasellar cysts; a taper was initiated but a transient increase in corticosteroids was required to control recurring symptoms. He was finally tapered off of corticosteroids on 10/19/2002 but the next day suffered acute right leg weakness and a facial droop. MRI showed a new infarct. On our suggestion, he was restarted on 12 mg dexamethasone/day and albendazole and referred to NIH on 11/7/2002. At this time, he only complained of corticosteroid side effects; his neurological examination was normal. MRI of the brain revealed multiple infarcts in both hemispheres, enhancement of the suprasellar space and cerebral peduncles. Albendazole was continued and a taper of corticosteroids was suggested to his referring neurologist but was delayed. Methotrexate was added 1/23/03 for corticosteroid sparing and maintained as an anti-inflammatory agent to prevent additional infarcts. After albendazole and corticosteroids were stopped in March and July of 2003, respectively, he was followed with serial LPs and CSF evaluations. Approximately 5 years after stopping therapy, an increase in CSF WBC count was documented along with an increased CSF cestode antigen level. The patient was asymptomatic. The brain MRI was unchanged but 2 small nodular enhancing lesions of unclear significance were noted in the lumbar spine. Treatment with albendazole and low dose corticosteroids were restarted and subsequently the WBC count reverted to normal

and the cestode antigen level became repeatedly undetectable. One of the spinal lesions regressed. After stopping treatment, he remains asymptomatic to the present over 14 years later. He underwent multiple joint replacement as a side effect of corticosteroids.

Patient 20

The patient is a 37-year-old male from El Salvador first seen at NIH 7/12/07. He was well until November 2006 when he developed progressive lower extremity weakness, band-like abdominal pain around T5 and difficulty urinating. He had spinal compression from racemose cysts and underwent laminectomy and resection of subarachnoid cysts at T3-5. At that time, he also was found to have extensive brain involvement. He was started on dexamethasone and albendazole but was lost to follow up. About 2 months prior to his second hospitalization, he began to have occipital headaches and left arm weakness. MRI showed massive involvement including envelopment and compression of the medulla by a cystic mass, extensive cystic enlargement of multiple basal cisterns including the cisterna magna and bilateral Sylvian fissure cysts. In addition, there were high cervical spine cysts with impending compression of the spine. He underwent debulking posterior craniotomy and spinal surgeries in early July 2007. His extensive treatment course, too long and complicated to give in detail, is outlined. In summary he was given multiple courses of treatment from July 2007 until late August 2012. He responded well to treatment and became asymptomatic in response to courses of treatment with either albendazole or praziquantel or the combination of both drugs along with

methotrexate and corticosteroids. These were tapered to a low dose and then stopped after resolution of all cysts and stable serial MRIs of the brain and spine. However, serial CSF analyses including CSF cestode antigen levels remained abnormal. WBC counts were variable ranging from roughly 98-35 WBC/mm^3 and cestode antigen levels were consistently extremely elevated above the limits of the assay. Because of uncertainty of the interpretation of extraordinarily high the cestode antigen levels in the setting of unchanged consolidated MRI imaging of brain and spine, and his asymptomatic status, all treatments were stopped, and he was briefly lost to follow up. On 11/18/15 the patient was still asymptomatic, but MRI showed regrowth of para-medullary cysts and a continued high cestode antigen. A number of empiric regimens were tried including high dose praziquantel with delayed administration of corticosteroids followed by a rapid taper off corticosteroids and then high dose praziquantel (about 100 mg/kg/day) combined with standard dose of albendazole without immunosuppressive medications. The medulla lesions, which had regrown while off therapy quickly degenerated. Although he developed a brief acute aseptic meningitis episode at the time of high-dose praziquantel without corticosteroids, which indicated treatment effects, the cestode antigen levels remained at very high levels. More recently, he was started on high dose albendazole (about 30 mg/kg/day), a moderate dose of praziquantel due to gastrointestinal intolerance without immunosuppressive medications. On this regimen cestode antigen levels have decreased dramatically accompanied by a moderate decrease of CSF WBC numbers. The patient continues to be asymptomatic with low but decreasing cestode CSF antigen levels, serially unchanged MRI imaging of brain and spine while off of all immunosuppressive medications.

Patient 26

The patient is a 34-year-old female with a prolonged undiagnosed course of SUBNCC. When she was 28 years of age, while residing in Guatemala, she started having severe headaches. At about the same time she suffered a stroke causing right-sided paralysis, which resolved over several months. The patient also related a vague history of prior seizures. She immigrated to the U.S. in July of 2004 and sought medical help after developing increasingly severe headaches, nausea and vomiting on 10/13/05. MRI showed extensive SUBNCC, an old left basal ganglion lacunar infarct resulting in ex vacuo hydrocephalus on the left as well as mild hydrocephalus on the right. She had cystic involvement of the right and left perimesencephalic spaces, middle cranial fossa on the right apparently from extension from a cyst in the right perimesencephalic cistern, prepontine cysts and localized enhancement associated with some of the lesions. LP showed 13 WBC/mm^3, mostly lymphocytes and 15% eosinophils. On 10/15/05 she was started on high-dose dexamethasone followed on 10/17/05 by albendazole. She was first evaluated at NIH on 10/24/05. Methotrexate was added to the regimen on 11/7/05. Thereafter, the patient was frequently confused and ingestion of medication erratic, so the cause of her varying symptoms was hard to interpret. Eventually the patient was able to take her medications reliably and clinically stabilized. Nevertheless, while tapering corticosteroids and still on cysticidal medications, she developed worsening hydrocephalus and headaches requiring a ventriculoperitoneal shunt insertion on 2/8/06. The patient considerably improved while undergoing a corticosteroid taper and continued cysticidal treatment. By July of 2006 the

MRI had stabilized, all cystic lesions resolved, and the patient was asymptomatic. Albendazole, dexamethasone and methotrexate were stopped on 3/22/06, 7/1/06, and 4/10/06, respectively. She felt well and decided to return to Guatemala in May 2008 and was then lost to follow up.

Patient 27

The patient is a 44-year-old female who immigrated to the US in 1986 when 13 years of age. In 1991 (17 years of age) the patient was diagnosed and treated for a pituitary adenoma, which was surgically removed. At that time, she was told she had neurocysticercosis and given a prescription for medication, which she did not take because of the expense. In 2003 she complained of headaches and episodes of numbness and weakness on the left side of her body. Weakness started on the left side with shaking of her arms progressing to her legs and then generalized weakness. An MRI documented cystic changes in the left and right perimesencephalic spaces that progressed since 1991 consistent with NCC. Headaches worsened and in 2011 she experienced transient episodes of unconsciousness, nausea and vomiting. Worsening headaches and blurred vision led to hospitalization in November of 2011. MRI showed massive SUBNCC of most of the basilar cisterns, cistern magna and bilateral Sylvian fissures resulting in hydrocephalus. A ventriculoperitoneal shunt was inserted and albendazole and steroids initiated in November of 2012 resulting in significant decrease in the size of the cysts and decreased hydrocephalus. The patient lives and works in Eastern North Carolina

where she receives most of her care. Because of time constraints evaluations at NIH have been limited. She was first seen at NIH in July of 2013. She had had 4 episodes of transient hemiparesis over the past 6 years but since the start of therapy her symptoms had improved and at NIH she only complained of dizziness. Her only neurological abnormality was a positive Romberg test. She was taking albendazole and 5 mg prednisone daily, which were continued. Because of the history of transient hemiparesis episodes, elevated CSF WBC counts suggesting persistent inflammation, and prolonged use of corticosteroids with significant side effects, methotrexate was added to her treatment regimen. She did reasonably well over the short term. Albendazole and corticosteroids were stopped on 12/7/13 after the CSF cestode antigen level became undetectable. MRI showed no cysts and consolidated stable disease. However, in March 2014 while at home she experienced another transient episode of hemiparesis that was not further evaluated. On 3/20/14 she was restarted on high-dose dexamethasone and etanercept while continuing methotrexate. She dramatically improved and all medications were stopped except methotrexate, which was continued until 9/11/14. Over the next few years off of all medications except for an anti-depressive drug, she had a single transient episode of symptoms lasting 3 days consisting of left-sided numbness, headaches and blurry vision from which improved over 3 days. Shortly thereafter she experienced a transient episode of generalized paralysis and aphasia, which again resolved after a few days. She declined further medical evaluations for these and related them to us retrospectively. In 2017 she complained of frequent symptoms of dizziness and headaches and on and off symptoms of depression but no further symptoms involving her left side. In April 2019 she had stopped all medications. Her symptoms were stable if not somewhat improved, but she experienced about a 1.5-hour

episode consisting of left sided rigidity and paresthesias along with tongue numbness. She continued to complain of intermittent severe headaches and symptoms of depression. However, she is able do considerable house work and take care of her family. She was restarted on anti-depressive medication. MRIs including studies shortly after her neurological episodes have been unchanged since first seen at NIH in 2013.

Patient 31

The patient is a 26-year-old Hispanic male who sustained a crush injury to his left arm and neck in March 2008. During evaluation for continued arm pain, MRI imaging of the brachial plexus and neck revealed an asymptomatic 4th ventricular cyst, which was uneventfully removed in early December 2009. He was first seen at NIH on 12/23/09. A post-surgical LP showed 4 WBC/mm^3 with normal protein and glucose values and low positive cestode CSF antigen. An asymptomatic right Sylvian fissure cyst was incidentally detected as part of his initial NIH evaluation. Treatment was delayed because of the contemplated arm surgery. He was started on corticosteroids and albendazole and corticosteroids on 7/30/10, leading to resolution of the Sylvian fissure cyst; he remained asymptomatic. Albendazole was stopped on 9/12/10 and corticosteroids on 10/25/10. No post treatment LPs were done. He has recovered complete use of his arm and remains asymptomatic without recurrence to the present.

Patient 33

The patient is a 45-year-old Hispanic male with biopsy proven SUBNCC and a second undefined histoproliferative process involving the spine and suprasellar region. The latter is responsible for practically all his symptoms. He migrated from El Salvador 27 years earlier and has returned yearly since 2005. He was well until August of 2011 when he developed a cervical lesion resulting in right arm rigidity progressing to paralysis. The lesion involving C4 was excised. It consisted of lymphocytes, plasma cells and granulomas; no organisms were identified, and no specific diagnosis was determined. After cervical surgery he was well until April 2012 when he developed blurry vision in the left eye. MRI showed masses involving the suprasellar and sellar regions and prepontine cistern, bilateral internal auditory canals, and right cerebellopontine angle. Biopsies obtained from exploratory surgery on 12/12/13 proceeding from the right peripheral Sylvian fissure medially showed cestode tegument peripherally and a lymphocytic plasma cell-containing mass within the sellar/suprasellar region. Neither identifiable parasite tissues nor *T. solium* DNA (subsequent analysis on fixed tissue) were detected in the biopsied sellar/suprasellar masses. He was given a short course of albendazole, presumably with corticosteroids starting on 1/2/13 ending on 1/16/13. He was first seen at NIH on 9/12/13. His physical examination was normal except for neurological evaluation which revealed he had lost most of the sight in his left eye except the superior nasal quadrant. By December of 2013 the cervical spine lesion had regrown eventually progressing to involve C1-7 ventrally and C2-7 dorsally. It displaced the spinal cord but did not cause symptoms. The diagnosis of NCC was confirmed by review of the brain biopsies, and positive Western blot for *T. solium*. LP on 9/18/13 showed 106/mm^3 WBC, 94% lymphocytes, 116 mg/dl protein, 55 mg/dl glucose, and

positive CSF cestode antigen. Initially, we thought that the proliferative process was in some way related to NCC, but the clinical and radiologic course and molecular studies did not support this hypothesis. Rosai-Dorfman disease was suggested as a possible diagnosis but could not be proven. Since he clearly had SUBNCC, he was treated with high dose corticosteroids from 11/12/13 until 5/1/14, albendazole from 11/21/13 to 4/3/14 and praziquantel from 1/9/14 to 4/1/14. There was little change in the masses and the patient was clinically stable. He remained clinically stable but developed acute hearing loss of unclear cause on 6/14/14 and was treated with high-dose steroids for about 2 weeks with full recovery. Around 5/21/15 both the cervical and brain masses had increased in size and surgery scheduled. At the patient's request surgery was postponed until after his yearly trip to El Salvador. He returned in early September 2015 but just before travel, while at the airport, he developed acute vision loss in his right eye. He was started on high dose steroids although vision loss occurred some days earlier. Surgery on 9/8/15 for decompression, debulking and rebiopsy showed brain CSF with 22/mm^3 WBC, 86% lymphocytes. Lumbar CSF on 9/10/15 showed 1 WBC/mm3, cestode antigen undetectable. Analysis of unfixed tissue was again inconclusive, and the diagnosis remained elusive. The molecular and histopathological studies of the mass did not support a diagnosis of a brain tumor and no parasite DNA was detected. He regained some vision in his right eye. The patient was referred elsewhere for treatment of the histoproliferative process.
