# Supplementary material for: Natural History of Treated Subarachnoid Neurocysticercosis
Source: Am J Trop Med Hyg. 2019 Oct 21;102(1):78–89. doi: 10.4269/ajtmh.19-0436 (PMC6947806; doi:10.4269/ajtmh.19-0436)
Supplement: Supplementary file 2 [file tpmd190436.SD2.pdf]

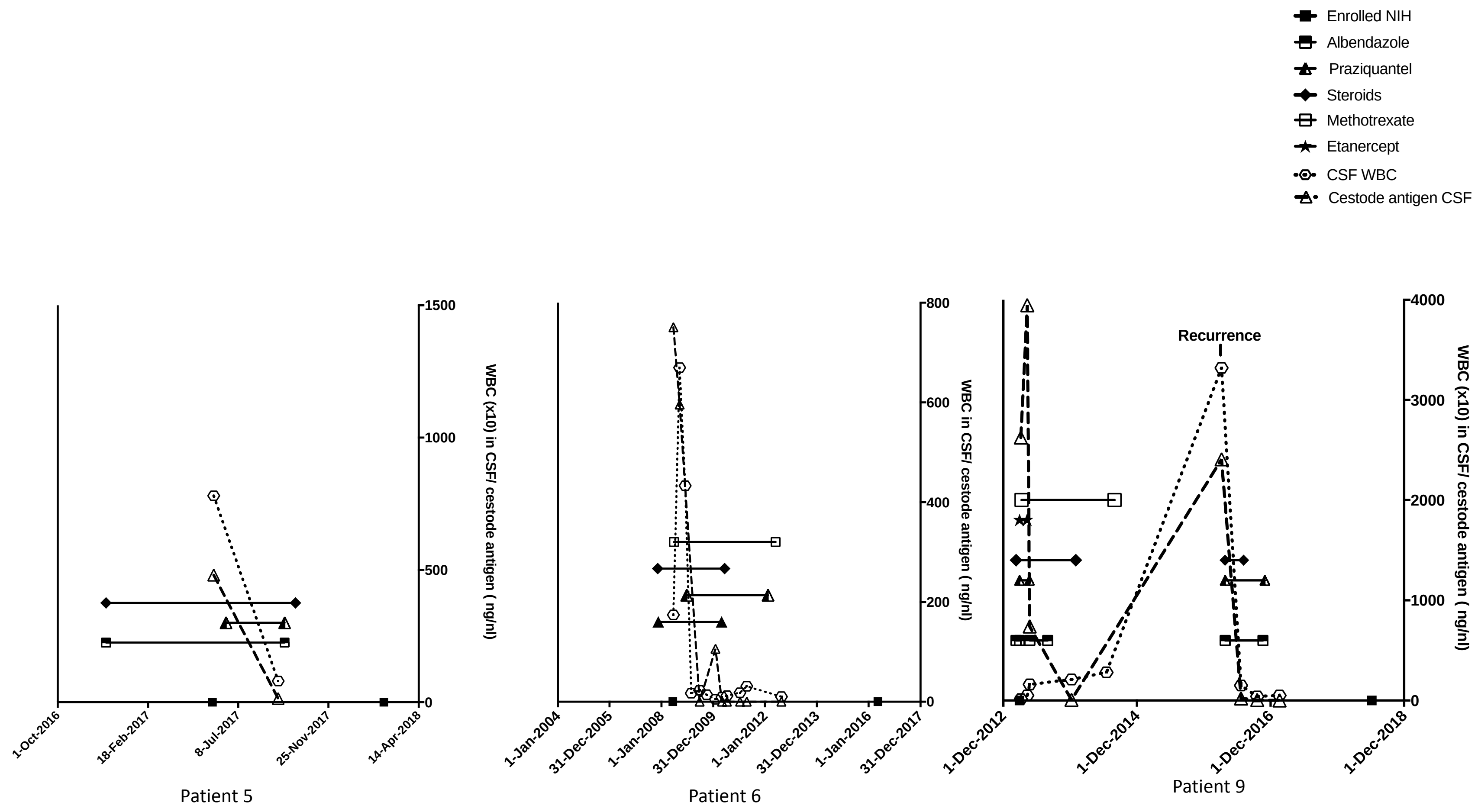

Supplemental Figure 5. Course of infection, treatment and follow up

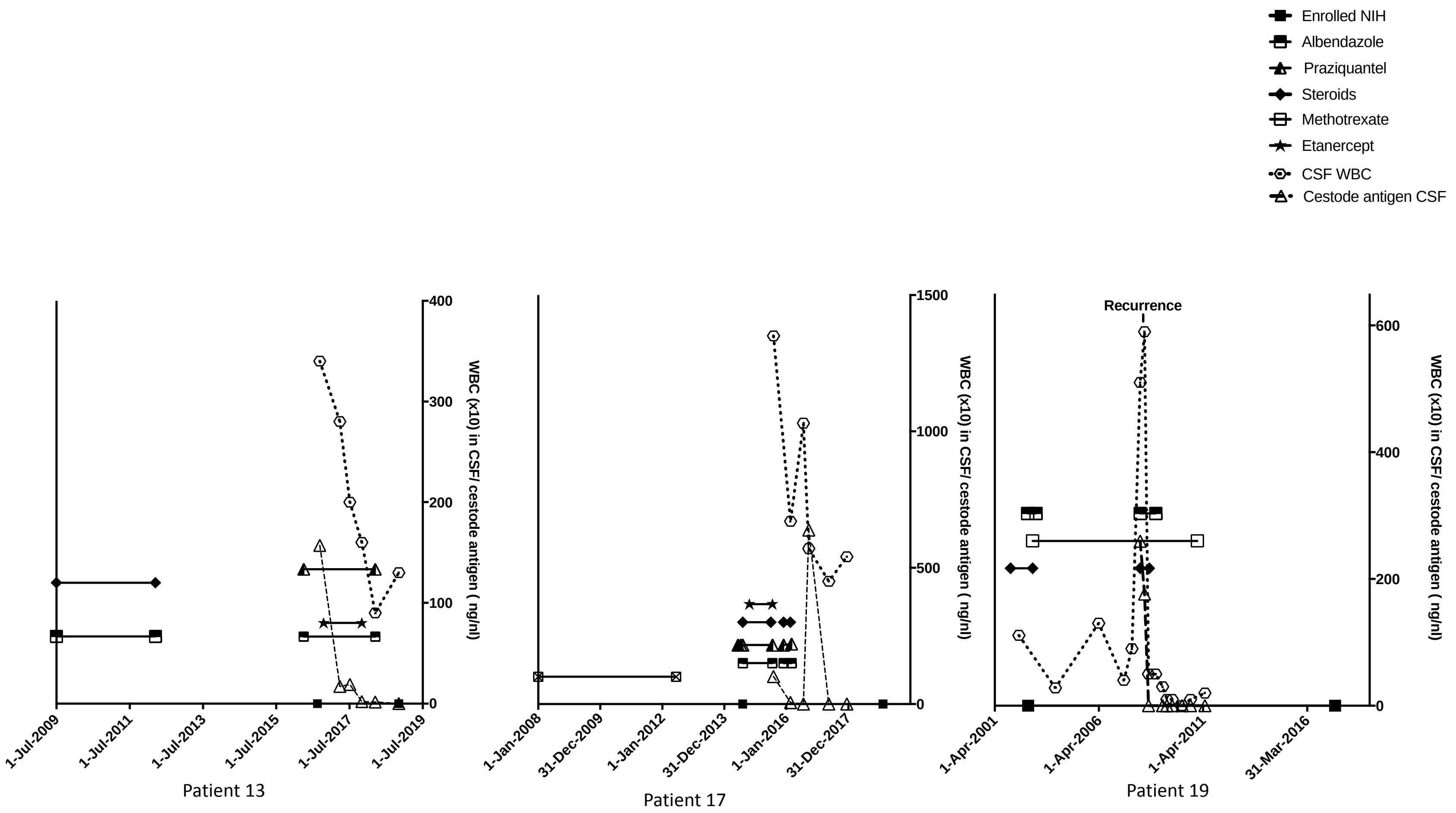

Supplemental Figure 6. Course of infection, treatment and follow up

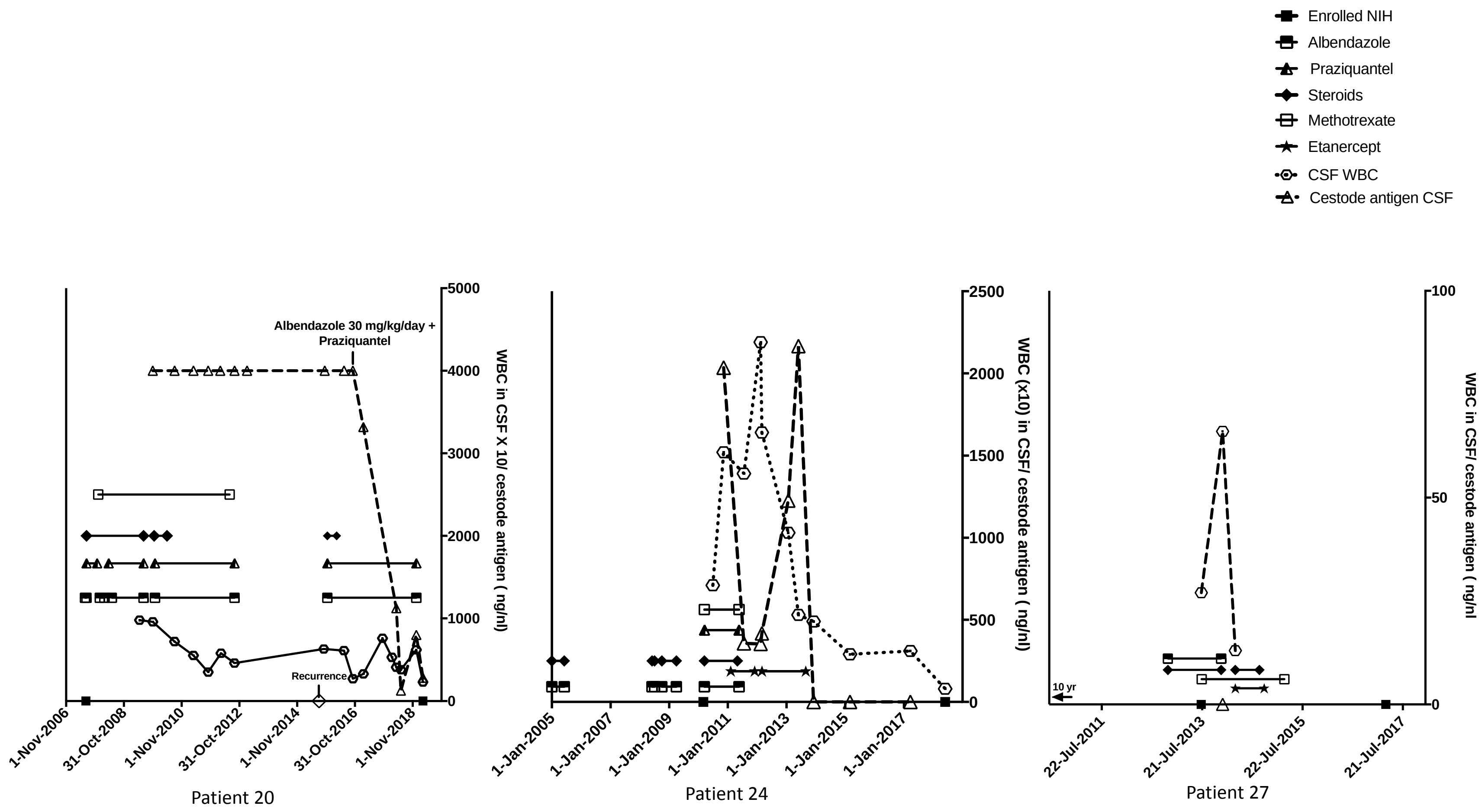

Supplemental Figure 7. Course of infection, treatment and follow up

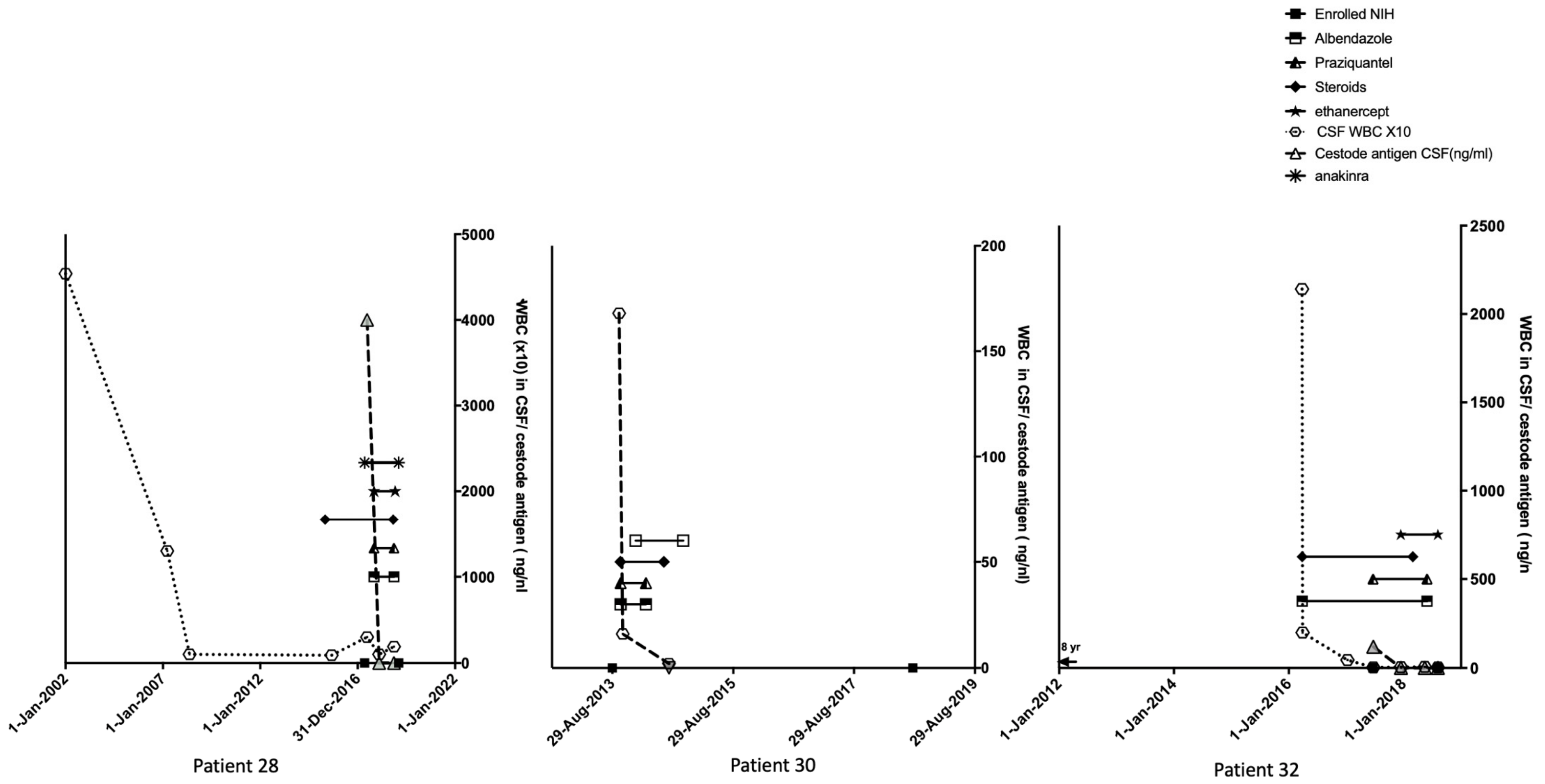

Supplemental Figure 8. Course of infection, treatment and follow up.

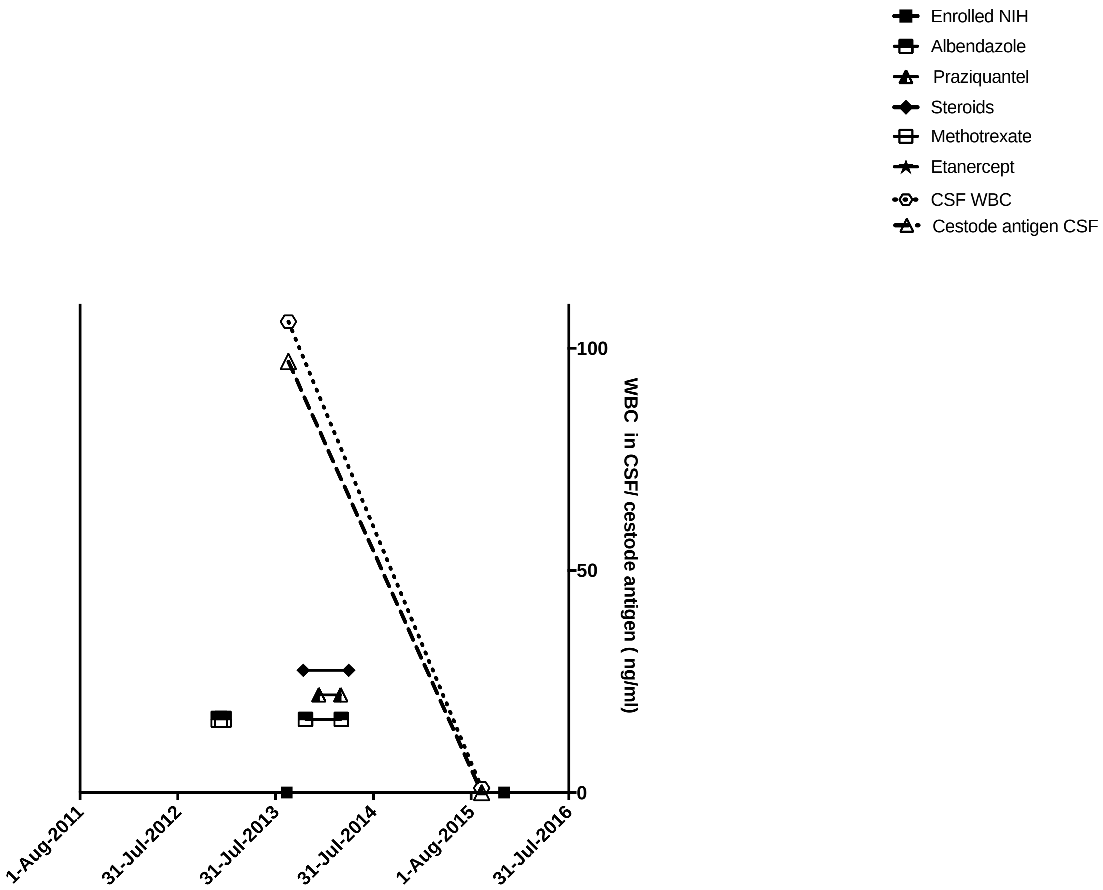

Patient 33

Supplemental Figure 9. Course of infection, treatment and follow up
